# The Circulating CTRP13 in Type 2 Diabetes and Non-Alcoholic Fatty Liver Patients

**DOI:** 10.1371/journal.pone.0168082

**Published:** 2016-12-09

**Authors:** Mehrnoosh Shanaki, Reza Fadaei, Nariman Moradi, Solaleh Emamgholipour, Hossein Poustchi

**Affiliations:** 1 Department of Laboratory Science, School of Allied Medical Science, Shahid Beheshti University of Medical Sciences, Tehran, Iran; 2 Department of Biochemistry, Faculty of Medicine, Tehran University of Medical Sciences, Tehran, Iran; 3 Department of Biochemistry, Faculty of Medicine, Iran University of Medical Sciences, Tehran, Iran; 4 Liver and Pancreatobiliary Diseases Research Center, Digestive Diseases Research Institute, Tehran University of Medical Sciences, Tehran, Iran; University of Colorado Denver School of Medicine, UNITED STATES

## Abstract

Numerous studies have shown that C1q/TNF-related proteins (CTRPs) are involved in the pathophysiology of metabolic disorders, such as Non-alcoholic fatty liver disease (NAFLD) and Type 2 Diabetes (T2DM). There is a little information concerning CTRP13 in the context of NAFLD and T2DM. We evaluated the plasma levels of CTRP13 in healthy control and patients with NAFLD, T2DM and NAFLD+T2DM, and also correlations between CTRP13 plasma levels and clinical and subclinical features. Circulating CTRP13 was examined in 88 male (20 healthy control, 22 T2DM patients, 22 NAFLD patients and 22 NAFLD+T2DM patients). CTRP13 and adiponectin plasma levels were measured by ELISA method. CTRP13 serum levels were higher in the control group than the other groups (all p <0.001). CTRP13 had significant negative correlation with unfavorable anthropometric and metabolic factors including BMI, visceral fat, Insulin, HOMA-IR, TG, AST, ALT and ɣ-GT and have a positive correlation with plasma concentration of adiponectin. CTRP13 had a significant inverse correlation with cIMT (r = -0.345) and liver stiffness (LS) (r = -0.372) (both, p <0.001). Also, the multiple stepwise linear regression has shown that visceral fat is a significant predictor of CTRP13 serum levels (p <0.001). Multiple stepwise linear regression with LS as the dependent variable showed that ALT (p < 0.001) and SBP (p = 0.010) were two predictor factors for LS. Strikingly, multiple stepwise linear regression showed that CTRP13 (p = 0.006) and SBP (p = 0.007) were two independent predictors for cIMT. Lower CTRP13 in patients with T2DM, NAFLD and NAFLD + T2DM was associated with increased risk of the diseases. CTRP13 have negative associations with unfavorable metabolic factors and also is a negative predictor of cIMT. Our results suggested that CTRP13 could be an associated factor with NAFLD in patients with and without T2DM.

## Introduction

Nonalcoholic fatty liver disease (NAFLD) is a spectrum of progressive liver disease which includes simple steatosis, NASH, fibrosis and eventually cirrhosis [1]. Several studies have indicated that NAFLD has a significant relationship with the features of metabolic syndrome, such as abdominal obesity, glucose intolerance or diabetes mellitus type 2 (T2DM) and insulin resistance [2]. Over the last few decades, NAFLD has become the most common cause of liver disorders in various race⁄ethnicity [3]. This is mainly because of dietary change and lifestyle behaviors which lead to increased rates of obesity and insulin resistance [3]. High prevalence of NAFLD has made it an important public health problem in many countries [4]. Understanding the pathobiology of NAFLD is essential for developing effective treatment and prevention strategies. However, the pathobiology mechanisms by which NAFLD initiates and progresses are not fully understood. In recent years, several tissue-based markers have been described for NAFLD, examples being C1q/TNF-related proteins (CTRPs) [5,6]. CTRPs are a new family of adipokines with 15 members (CTRP1 to CTRP15). This protein family shares a common structure but can be different in regulation and function [7].

CTRP13 has been known as a highly conserved member of CTRP family and improves insulin sensitivity as well as lipid induced stress on insulin signaling and glucose uptake. Subsequently, it was found that this protein decreases gluconeogenesis [8]. CTRP13 consist of C-terminal globular domain homologous to the immune complement protein C1q, two highly conserved Cys residues and 17 Gly-X-Y repeats in collagen domain. In human, most of CTRP13 was expressed by adipose tissue and secreted as a multimeric protein [8]. This is also worth mentioning that caloric restriction decreases, and high calorie diet increases expression level of CTRP13 mRNA in brain tissue of mice [9]. However, little information is available concerning CTRP13 in the context of NAFLD and T2DM. We have previously describe the effect of NAFLD and T2DM on plasma levels of CTRP1 and CTRP5 [5,10]. Here, we aimed to evaluate the plasma levels of CTRP13 in healthy control and patients with NAFLD, T2DM and NAFLD+T2DM. Additionally, possible associations with anthropometric, metabolic parameters and subclinical atherosclerosis were examined.

## Study subjects and methods

### Patients and Controls

A total of 88 male (20 healthy control, 22 T2DM patients, 22 NAFLD patients and 22 NAFLD+T2DM patients) were recruited from Shariati Hospital from May 2014 to Nov 2015. All patients were newly diagnosed. T2DM was diagnosed based on American diabetes association (ADA) criteria [11]. NAFLD was diagnosed using ultrasonography and Liver Stiffness (LS) was determined by elastography. Also, carotid intima media thickness (cIMT) and the amount of visceral fat measured by ultrasonography.

Control subjects were randomly selected from the population and did not have any metabolic disorder. Patients and controls had no significant differences in ethnicity. All subjects provided written consent, and the study was approved by the ethics committee of Tehran University of Medical Science. Subjects who had evidence of special conditions were excluded from the study. These conditions include type 1 diabetes mellitus, acute or chronic renal failure, congenital cardiac disease, infectious disease, malignancies and other liver disease such as Wilson’s disease, primary biliary cirrhosis viral or autoimmune hepatitis and haemochromatosis and also a history of alcohol consumption (>30g/day).

### Anthropometric and laboratory measurement

Anthropometric parameters of study subjects, including age, height, weight and blood pressure, were measured and body mass index (BMI) also calculated using following equation: body weight (kg) divided by the square of height (m^2^). Five mL venous blood was collected from study participants after overnight fasting. Fasting blood sugar (FBG), triglyceride (TG), total cholesterol (TC), low density lipoprotein cholesterol (LDL-C), high density lipoprotein cholesterol (HDL-C), Creatinine (Cr), Urea, gamma glutamyl transferase (GGT), alanine amino transferase (ALT), aspartate amino transferase (AST), alkaline phosphatase (ALP) were measured by auto analyzer using commercial kits (Pars Azmoon, Tehran, Iran). Fasting plasma insulin was measured by ELISA kit (Monobind Inc., USA). Homeostasis Model Assessment of Insulin Resistance (HOMA-IR) were calculating with the following equation: [fasting blood sugar (mg/dL)] × [fasting blood insulin (μU/mL)] / 405.

### Ultrasonography and elastography

Ultrasonography was conducted for evaluating liver, visceral fat and cIMT, using Accuvix XQ ultrasound unit (Medison, seoul, South Korea) equipped with a 5–12 MHz linear-array transducer and a 3–7 MHz curved-transducer as previously described [12]. Also, we used average of the right and left mean IMT values as cIMT. LS was measured by FibroScan 502 machine (EchoSense, Paris, France, 5MHz) with X and XL probes according to manufacturer’s protocol [10]. We considered cIMT≥0.90 mm as a typical cutoff value for determine number of subjects with significant risk for coronary artery disease [13].

### Measuring plasma levels of adiponectin and CTRP13

Adiponectin plasma levels was measured using the ELISA Kit (Elabscience, Wuhan, China) according to manufacture protocol, Intra-assay Coefficients of Variability (CV) was <10% and Inter-assay CV was <10%. Plasma levels of CTRP13 was measured by ELISA kit (Cusabio Biotech Co, Wuhan, China) according to manufacturer’s protocol. Intra-assay and inter-assay CV were <8% and <10%, respectively.

### Statistical analysis

Continuous variable with normal distribution was shown with mean ± standard error of mean (SEM) and skewed distributed variables was shown with median (IQR). ANOVA with scheffe post hoc was performed for normal distributed data, and Kruskal wails and Bonferroni correction post hoc were used for non-normal distributed data. Then, ANCOVA analysis was performed to remove effect of potential confounder. Before correlation analysis, non-normal distributed data was logarithmic transformed, and Pearson’s correlations was used to evaluate correlation between CTRP13 with different anthropometric and laboratory variables. Also, we performed univariate linear regression with CTRP13, LS and cIMT as dependent variable. If variables reached statistical cut off (p value<0.2), included to multiple stepwise linear regression to identify significant predictor of CTRP13, LS and cIMT. Multinomial logistic regression was performed to identify risk of each condition (T2DM, NAFLD and NAFLD+T2DM) with regarding to CTRP13 plasma levels. ROC curve was plotted to evaluating sensitivity and specificity of CTRP13 plasma levels to differentiate between each condition. All analysis was conducted with SPSS 16 (SPSS, Chicago, IL, USA) and p<0.05 considered as statistical significant.

## Results

Clinical and laboratory characteristics of the study population were shown in Table 1. According to ANOVA, BMI, visceral fat, blood pressure, FBG, Insulin, HOMA-IR and adiponectin were different and post hoc analysis showed the significant difference were mainly between the control group and other groups. Also, factors related to measuring the function of the liver, including ALT, AST, GT-ɣ as well as the LS were increased in NAFLD and NAFLD + T2DM groups in compared to other groups. The cIMT in NAFLD + T2DM group was higher than the control group. Also, the number of patients with cIMT≥0.90 mm were higher in T2DM and NAFLD+T2DM groups compared with control and NAFLD groups (p<0.05). There was no significant difference in terms of: age, TG, TC, HDL-C, LDL-C, urea nitrogen, creatinine and ALP in four study groups.

**Table 1:** Anthropometric and laboratory characteristics of study subjects.

Variables	Control (20)	T2DM (22)	NAFLD (22)	T2DM+NAFLD (22)	P value
Age (years)	53.40 ± 1.81	57.36 ± 1.58	52.23 ± 1.29	52.86 ± 1.33	0.072
BMI (kg/m^2^)	24.92 ± 0.82	27.11 ± 0.81	29.34 ± 0.79^b^**	30.69 ± 0.69^c^***	<0.001
Visceral Fat (mm)	44.45 ± 3.82	62.14 ± 4.09^a^*	70.82 ± 4.13^b^***	78.27 ± 4.31^c^***	<0.001
SBP (mmHg)	122.35 ± 3.92	136.27 ± 4.38	135.18 ± 3.28	142.23 ± 4.44^c^*	0.009
DBP (mmHg)	77.30 ± 2.44	79.95 ± 2.20	84.77 ± 2.49	85.59 ± 1.92	0.036
FBG (mg/dL)	89.9 (83.4–97.0)	150.20 (123.1–191.6)^b^***	96.5 (87.2–101.7)^d^***	162.5 (128.2–187.1)^c^***^,^^f^***	<0.001
Insulin (μU/mL)	3.20 (1.32–6.01)	6.65 (2.45–9.19)	8.65 (7.12–12.15)^b^**	7.10 (4.58–11.04)^c^***	<0.001
HOMA-IR	0.83 ± 0.13	2.70 ± 0.35^a^**	2.27 ± 0.25^b^*	3.40 ± 0.45^c^***	<0.001
TG (mg/dL)	111.9 (90.8–156.1)	146.4 (104.4–166.3)	140.6 (93.7189.8)	154.3 (113.8–196.0)	0.099
TC (mg/dL)	184.07 ± 6.59	204.74 ± 10.65	203.71 ± 7.21	209.06 ± 7.80	0.197
HDL-C (mg/dL)	52.21 ± 2.40	54.18 ± 2.59	51.55 ± 2.76	55.46 ± 2.35	0.688
LDL-C (mg/dL)	108.26 ± 6.54	122.57 ± 9.49	121.00 ± 5.18	127.50 ± 9.20	0.388
Urea nitrogen (mg/dL)	28.89 ± 1.22	31.76 ± 0.96	29.76 ± 1.36	32.63 ± 1.40	0.141
Creatinine (mg/dL)	1.22 ± 0.06	1.27 ± 0.05	1.27 ± 0.03	1.26 ± 0.06	0.905
AST (U/L)	18.0 (15.2–20.4)	16.4 (14.6–21.7)	25.4 (20.3–33.3)^b^**^,^^d^**	23.0 (20.0–28.2)^c^*^,^^e^*	<0.001
ALT (U/L)	16.2 (13.4–21.4)	17.6 (13.4–27.3)	31.6 (22.2–44.2)^b^**^,^^d^*	38.2 (23.6–50.4)^c^***^,^^e^**	<0.001
ALP (U/L)	222.8 ± 11.2	257.9 ± 16.8	217.6 ± 12.0	231.0 ± 13.3	0.154
ɣ-GT (U/L)	18.3 (15.6–20.8)	23.9 (20.4–36.7)^a^*	32.7 (21.7–43.8)^b^***	37.0 (27.9–60.5)^c^***	<0.001
LS (kPa)	3.78 ± 0.24	4.08 ± 0.37	6.30 ± 0.28^b^***^,^^d^***	7.25 ± 0.29^c^***^,^^e^***	<0.001
cIMT (mm)	.76 ± 0.02	0.84 ± 0.02	0.80 ± 0.02	0.88 ± 0.03^c^*	0.006
cIMT≥0.90mm [n (%)]	1 (5)	6 (27.3)	2 (9.1)	10 (45.5)	0.005
Adiponectin (ng/mL)	4.90 ± 0.34	3.61 ± 0.30^a^*	2.98 ± 0.24^b^***	3.59 ± 0.32^c^*	<0.001
CTRP13 (ng/mL)	3.67 ± 0.24	2.37 ± 0.21^a^***	2.10 ± 0.18^b^***	1.75 ± 0.15^c^***	<0.001

a: Comparison between Control and T2DM

b: Comparison between Control and NAFLD

c: Comparison between Control T2DM-NAFLD

d: Comparison between T2DM and NAFLD

e: Comparison between T2DM and T2DM+NAFLD and

f: Comparison between NAFLD and T2DM+NAFLD.

*P < 0.05

**P < 0.01

***P < 0.001.

**Fig 1:**
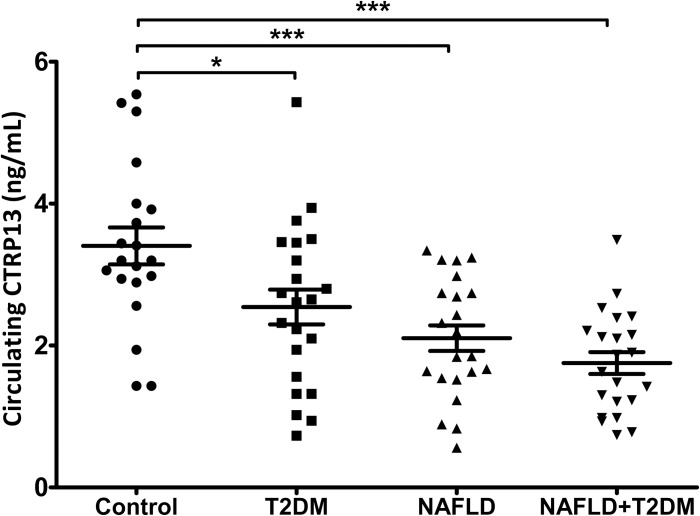
Circulating CTRP13 in study subjects. Data present with mean+SEM. * and *** represent p<0.05 and p<0.001, respectively.

CTRP13 serum levels were higher in the control group than the other groups (all p <0.001), as shown in Fig 1. ANCOVA analysis was performed to eliminate the effect of age, BMI and adiponectin and it was found that a significant difference for T2DM group than the control group had disappeared, but the difference remained significant between the control group and NAFLD and NAFLD + T2DM groups (Table 2).

**Table 2:** A full factorial model of ANCOVA to adjust the effect of age, BMI and adiponectin on CTRP13 plasma levels in healthy controls, T2DM, NAFLD and NAFLD+T2DM patients.

(I) Group	(J) Group	Mean Difference (I-J)	Std. Error	Sig.^b^	95% Confidence Interval for Difference^b^	
					Lower Bound	Upper Bound
HC	T2DM	.711	.344	.252	-.220	1.641
	NAFLD	1.168*	.366	.012	.178	2.159
	NAFLD+T2DM	1.520*	.371	.001	.515	2.526

*. The mean difference is significant at the 0.05 level.

b. Adjustment for multiple comparisons: Bonferroni.

Bivariate correlation analysis of CTRP13 with anthropometric and laboratory parameters was shown in Table 3. CTRP13 had significant negative correlation with unfavorable anthropometric and metabolic factors including BMI, visceral fat, Insulin, HOMA-IR, TG, AST, ALT and ɣ-GT and have a positive correlation with plasma concentration of adiponectin. After adjusting for adiponectin concentration, all correlation remains significant except for negative correlation with TG. Interestingly, CTRP13 had a significant inverse correlation with cIMT (r = -0.345) and LS (r = -0.372) (both, p <0.001). The subgroup analysis showed, CTRP13 in diabetic patients correlated with visceral fat (r = -0.497, p = 0.024) and FBG (r = -0.550 and p = 0.008). Also, the multiple stepwise linear regression has shown that visceral fat is a significant predictor of CTRP13 serum levels (β [SE] = -0.022 [0.005], p <0.001). Multiple stepwise linear regression with LS as the dependent variable showed that ALT (β [SE] = 5.395 [0.917], p < 0.001) and SBP (β [SE] = 0.030 [0.011], p = 0.010) were two predictor factors for LS (S1 Table). Strikingly, multiple stepwise linear regression showed that CTRP13 (β [SE] = -0.028 [0.010], p = 0.006) and SBP (β [SE] = 0.002 [0.001], p = 0.007) were two independent predictors for cIMT (S2 Table).

**Table 3:** Correlation between CTRP13 plasma levels with anthropometric and laboratory variables.

	Unadjusted	Adjusted for adiponectin
Variables	CTRP13	CTRP13
Age	-.034	-.060
BMI	-.290**	-.231*
Visceral Fat	-.440**	-.414**
SBP	-.103	-.080
DBP	-.183	-.132
FBG^a^	-.148	-.131
Insulin^a^	-.315**	-.278*
HOMA-IR	-.225*	-.228*
TG^a^	-.239*	-.204
TC	.048	.051
HDL-C	.191	.196
LDL-C	-.014	-.046
Urea nitrogen	.018	.028
Creatinine	.082	.076
AST^a^	-.286**	-.267*
ALT^a^	-.270*	-.237*
ALP	.035	.008
ɣ-GT^a^	-.342**	-.291**
Liver Stiffness	-.372**	-.333**
cIMT	-.345**	-.339**
Adiponectin	.255*	---

a: Logarithmic transformation was performed

*p<0.05 and

**p<0.01.

**Fig 2:**
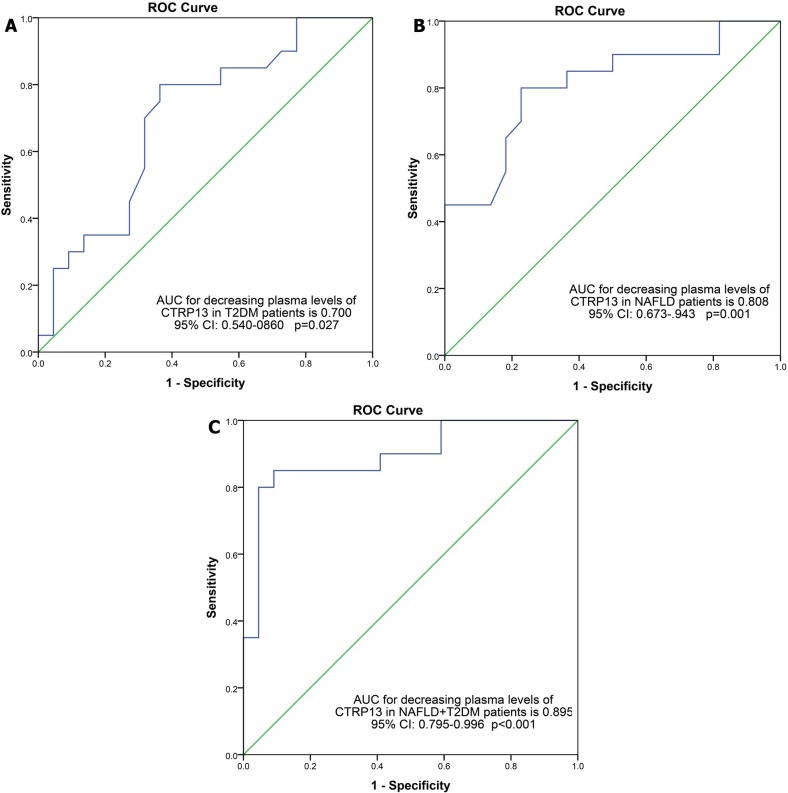
ROC curves for diagnosis NAFLD (A), T2DM (B) and NAFLD+T2DM (C), according to circulating CTRP13.

In addition, based on multinomial logistic regression, increased CTRP13 was associated with reduce the risk of T2DM, NAFLD and NAFLD + T2DM (Table 4). After adjustment for age, BMI and serum levels of adiponectin, this association remained significant, but in subjects with diabetes, association was disappeared after adjusting for age and BMI. ROC Curve data for evaluation of diagnostic value of CTRP13 also showed that the area under the curve were 0.700 for T2DM, 0.808 for NAFLD, and 0.895 for NAFLD + T2DM (as shown in Fig 2). This measurement approved the good specificity and sensitivity of CTRP13 as diagnostic biomarker.

**Table 4:** Multinomial logistic regression for odds ratios of T2DM, NAFLD and NAFLD+T2DM according to Circulating CTRP13.

		T2DM	NAFLD	NAFLD+T2DM
Model-1	B (SE)	-.762 (0.332)	-1.260 (.382)	-1.740 (.426)
	OR (CI)	0.467 (0.243–0.895)	0.284 (0.134–0.599)	.176 (0.076–0.404)
	p	.022	.001	< .001
Model-2	B (SE)	-.600 (0.337)	-1.119 (0.399)	-1.605 (0.471)
	OR (CI)	.549 (0.284–1.061)	0.327 (0.149–0.714)	.201 (0.080–0.505)
	p	.074	.005	.001
Model-3	B (SE)	-.502 (0.361)	-1.039 (0.432)	-1.558 (0.483)
	OR (CI)	.605 (0.298–1.229)	.354 (0.152–0.825)	.211 (0.082–0.543)
	P	.165	.016	.001

Model-1: unadjusted

Model-2: adjusting for age and BMI

Model-3: adjusting for age, BMI and adiponectin

## Discussion

CTRP13 plays an important role in regulating glucose metabolism and insulin sensitivity. Recent studies indicate the importance of the CTRP family in metabolic diseases[14], but so far there is no data about the role of CTRP13 in NAFLD. In our study, we have demonstrated that CTRP13 is lower in three patients groups (T2DM, NAFLD and NAFLD+T2DM) compared to the control group. This difference remained significant even after adjustment for age, BMI and serum adiponectin for NAFLD and NAFLD+T2DM groups. Our result is inconsistent with observations reported by Peterson JM et al and Byerly MS et al. They found that plasma level of CTRP13 decreased in in leptin-deficient mice [8,9]. This discrepancy may arise due to differences in sample type. It worth also mentioning that our data is inconsistent with some investigations that indicated the high plasma level of CTRP1 in NAFLD and T2DM [10]. This might be a possible explanation for the compensatory role of CTRP1 which increased in coronary artery disease and T2DM patients [15–17].

There is also several of evidence from preclinical investigations showing that CTRP3 have decreased in obese patients [18] and newly diagnosed T2DM [19]. Other studies have also shown that CTRP5 have decreased in NAFLD and T2DM patients [5]. It seems that our data support this view that CTRPs have reduced in plasma of NAFLD patients. In this *in vivo* study, we have demonstrated that serum levels of CTRP13 have a significant negative correlation with insulin and HOMA-IR. Our study is consistent with previous investigations, which showed that CTRP3 [20] and CTRP5 [5] negatively correlated with HOMA-IR in T2DM and obese, T2DM and NAFLD patients. In addition, CTRP13 showed a significant negative correlation with TG. According to the previous studies, AMPK function leads to beta-oxidation induction [21]. Since, AMPK can be induced by CTRP13 [8]; it seems that reduced CTRP13 serum levels have a negative effect on beta oxidation. However, molecular mechanistic studies are required to establish this concept.

In recent years, there has been an increasing number of studies in the literature on the correlation of adiponectin with BMI and visceral fat [22,23]. We showed that visceral fat is a significant negative predictor of plasma CTRP13, so that visceral fat may be associated with decreased serum levels of CTRP13. Also, it is believed that CTRP13 is expressed in adipose tissue [8] and appears to increase the volume of visceral adipose tissue associated with decrease circulating CTRP13. Unlike CTRP1, CTRP13 have a negative correlation with LS. The finding of our previous study indicated that CTRP1 has a positively correlation with LS and CTRP5 have a negative correlation with LS [5,10]. Compensatory role of CTRP1 which increased in NAFLD patients might be a possible explanation for the positive correlation of CTRP1 with LS. Negative correlation between CTRP13 and LS (as a marker for liver fibrosis [24]) needed further studies to prove possible casual relation between CTRP13 and liver fibrosis.

There is also a growing body of studies showing that CTRP1 and CTRP9 play a possible role in coronary artery disease and atherosclerosis [17,25,26]. In the present study we show that CTRP13 have a negative correlation with cIMT (as a marker for subclinical atherosclerosis), and CTRP13 also is a significant negative predictor of cIMT. Another study has indicated that adiponectin could be an additional independent factor associated with atherosclerosis [27]. Hence, it seems likely that decreasing CTRP13 serum levels associated with subclinical atherosclerosis, however more studies with a large sample size are necessary to establish this concept.

In current study, ROC curve analysis determined that CTRP13 has a good ability to distinguish between patients (T2DM, NAFLD and NAFLD + T2DM) and controls. These results suggest a potential marker role for CTRP13 in patients with NAFLD. However, future study with larger sample size on the current topic may be helpful.

This study has several limitations; first, sample size is relatively small to make a definite conclusion. Second, the case control design does not allow us to determine the causality between examined variables. Therefore, it is highly desirable to conduct further large-scale prospective clinical investigations in NAFLD. Also, all participant are Iranian population and our results should carefully be generalized to other populations. In addition, there is no study in human regarding role of CTRP13. Therefore, it makes difficult to compare and interpret our results with other studies.

In summary, we showed that lower CTRP13 in patients with T2DM, NAFLD and NAFLD + T2DM was associated with increased risk of the diseases. CTRP13 have negative associations with unfavorable metabolic factors and also is a negative predictor of cIMT. Our results suggested that CTRP13 could be an associated factor with NAFLD in patients with and without T2DM. Future study is needed to clarify the role of CTRP13 in pathogenesis of NAFDL and atherosclerosis.

## Supporting Information

S1 Table Univariate and multiple linear regression with LS as dependent variable. (DOCX)

S2 Table Univariate and multiple linear regression with cIMT as dependent variable. (DOCX)
