# Supplementary material for: The Circulating CTRP13 in Type 2 Diabetes and Non-Alcoholic Fatty Liver Patients
Source: PLoS One. 2016 Dec 9;11(12):e0168082. doi: 10.1371/journal.pone.0168082 (PMC5148106; doi:10.1371/journal.pone.0168082)
Supplement: S1 Table — (DOCX) [file pone.0168082.s001.docx]

**S1 Table. Univariate and multiple linear regression with LS as dependent variable.**

| **Univariate Linear Regression** | | | | | **Multiple Stepwise Linear Regression** | | | |
| --- | --- | --- | --- | --- | --- | --- | --- | --- |
| Variables | Unstandardized Coefficients | | Standardized Coefficients | Sig. | Unstandardized Coefficients | | Standardized Coefficients | Sig. |
|  | B | Std. Error | Beta |  | B | Std. Error | Beta |  |
| Age | .004 | .002 | .222 | .040 |  |  |  |  |
| BMI | .218 | .048 | .444 | .000 |  |  |  |  |
| SBP | .024 | .011 | .236 | .029 | .030 | .011 | .248 | .010 |
| DBP | .044 | .020 | .236 | .028 |  |  |  |  |
| Insulin^a^ | 1.351 | .460 | .305 | .004 |  |  |  |  |
| HOMA-IR | .370 | .119 | .320 | .003 |  |  |  |  |
| Urea | .065 | .038 | .187 | .095 |  |  |  |  |
| AST^a^ | 5.355 | 1.387 | .394 | .000 |  |  |  |  |
| ALT^a^ | 4.621 | .839 | .522 | .000 | 5.395 | .917 | .555 | .000 |
| ALP | -.006 | .004 | -.200 | .070 |  |  |  |  |
| ɣ-GT | 4.140 | .965 | .435 | .000 |  |  |  |  |
| Adiponectin | -.302 | .141 | -.228 | .035 |  |  |  |  |
| CTRP13 | -.657 | .179 | -.372 | .000 |  |  |  |  |

^a.^ Logarithmic transformation was performed.
