# Supplementary material for: The Circulating CTRP13 in Type 2 Diabetes and Non-Alcoholic Fatty Liver Patients
Source: PLoS One. 2016 Dec 9;11(12):e0168082. doi: 10.1371/journal.pone.0168082 (PMC5148106; doi:10.1371/journal.pone.0168082)
Supplement: S2 Table — (DOCX) [file pone.0168082.s002.docx]

**S 2 Table. Univariate and multiple linear regression with cIMT as dependent variable.**

| Univariate Linear Regression | | | | | Multiple Stepwise Linear Regression | | | |
| --- | --- | --- | --- | --- | --- | --- | --- | --- |
| Variables | Unstandardized Coefficients | | Standardized Coefficients | Sig. | Unstandardized Coefficients | | Standardized Coefficients | Sig. |
|  | B | Std. Error | Beta |  | B | Std. Error | Beta |  |
| Age | .004 | .002 | .222 | .040 |  |  |  |  |
| Visceral fat | .001 | .001 | .170 | .117 |  |  |  |  |
| SBP | .002 | .001 | .340 | .001 | .002 | .001 | .351 | .007 |
| DBP | .002 | .001 | .171 | .115 |  |  |  |  |
| FBG^a^ | .185 | .075 | .261 | .015 |  |  |  |  |
| LDL | .001 | .000 | .195 | .078 |  |  |  |  |
| ɣ-GT^a^ | .110 | .060 | .200 | .073 |  |  |  |  |
| LS | .013 | .006 | .237 | .028 |  |  |  |  |
| CTRP13 | -.034 | .010 | -.345 | .001 | -.028 | .010 | -.267 | .006 |

^a.^ Logarithmic transformation was performed
